# MEG8 regulates Tissue Factor Pathway Inhibitor 2 (TFPI2) expression in the endothelium

**DOI:** 10.1038/s41598-022-04812-z

**Published:** 2022-01-17

**Authors:** Veerle Kremer, Diewertje I. Bink, Laura Stanicek, Eva van Ingen, Theresa Gimbel, Sarah Hilderink, Stefan Günther, Anne Yaël Nossent, Reinier A. Boon

**Affiliations:** 1 Department of Physiology, Amsterdam Cardiovascular Sciences, VU Medical Center, Amsterdam UMC, De Boelelaan 1108, 1081 HZ Amsterdam, The Netherlands; 2 Department of Medical Biochemistry, Academic Medical Center, Amsterdam UMC, Amsterdam, The Netherlands; 3 Department of Surgery, The Netherlands Einthoven Laboratory for Experimental Vascular Medicine, Leiden University Medical Center, Leiden, The Netherlands; 4 Departments of Laboratory Medicine and Internal Medicine II, Medical University of Vienna, Vienna, Austria; 5 Institute of Cardiovascular Regeneration, Goethe University, Frankfurt am Main, Germany; 6 German Centre for Cardiovascular Research DZHK, Partner Site Frankfurt Rhein/Main, Frankfurt am Main, Germany; 7 Max Planck Institute for Heart and Lung Research, Bioinformatics and Deep Sequencing Platform, Bad Nauheim, Germany

**Keywords:** Cardiovascular biology, Cardiovascular diseases, Non-coding RNAs, Molecular biology

## Abstract

A large portion of the genome is transcribed into non-coding RNA, which does not encode protein. Many long non-coding RNAs (lncRNAs) have been shown to be involved in important regulatory processes such as genomic imprinting and chromatin modification. The 14q32 locus contains many non-coding RNAs such as Maternally Expressed Gene 8 (MEG8). We observed an induction of this gene in ischemic heart disease. We investigated the role of MEG8 specifically in endothelial function as well as the underlying mechanism. We hypothesized that MEG8 plays an important role in cardiovascular disease via epigenetic regulation of gene expression. Experiments were performed in human umbilical vein endothelial cells (HUVECs). In vitro silencing of MEG8 resulted in impaired angiogenic sprouting. More specifically, total sprout length was reduced as was proliferation, while migration was unaffected. We performed RNA sequencing to assess changes in gene expression after loss of MEG8. The most profoundly regulated gene, Tissue Factor Pathway Inhibitor 2 (TFPI2), was fivefold increased following MEG8 silencing. TFPI2 has previously been described as an inhibitor of angiogenesis. Mechanistically, MEG8 silencing resulted in a reduction of the inhibitory histone modification H3K27me3 at the TFPI2 promoter. Interestingly, additional silencing of TFPI2 partially restored angiogenic sprouting capacity but did not affect proliferation of MEG8 silenced cells. In conclusion, silencing of MEG8 impairs endothelial function, suggesting a potential beneficial role in maintaining cell viability. Our study highlights the MEG8/TFPI2 axis as potential therapeutic approach to improve angiogenesis following ischemia.

## Introduction

The endothelium is crucial for maintaining a healthy vasculature by controlling the exchange of fluids and molecules, the creation of new vasculature and regulation of vascular tone and blood pressure^1,2^. An important feature of endothelial cells (EC) is their capacity to sense and respond to angiogenic signals. These signals regulate the sprouting of vessels from pre-existing ones, a process called angiogenesis. The pro-angiogenic signal Vascular Endothelial Growth Factor (VEGF) induces activation of ECs, which become motile and invasive, forming the front of the vessel sprout. Migration of these so-called tip cells are guided by attractive or repulsive cues. In addition to tip cells, proliferative stalk cells form the vascular lumen of the new sprout. Finally, blood flow is essential for remodelling and maturation of the newly formed vessel^3,4^.

Multiple signalling pathways contribute to the regulation of angiogenesis. Recent studies have indicated a potential role for non-coding RNAs in this process^5^. Non-coding RNAs represent a class of transcripts which lack protein coding capacity and make up the majority of the transcriptome^6^. A major subgroup is known as long non-coding RNA (lncRNA), defined as non-coding transcripts with lengths greater than 200 nucleotides^7^. LncRNAs can localize throughout the nucleus and cytoplasm and are highly diverse in terms of function. Mechanistically, lncRNAs can interact with DNA, proteins and other RNAs or function as scaffolds to regulate various cellular processes. Cytoplasmic lncRNAs can be involved in posttranscriptional regulation. Many lncRNAs are reported to localize to the nucleus and modulate RNA splicing, transcription factor binding and chromatin structure^7–11^.

An important regulator of chromatin structure and transcription is the Polycomb Repressive Complex 2 (PRC2). PRC2 is a histone methyltransferase which catalyzes trimethylation at histone H3 lysine 27 (H3K27me3) to induce chromatin remodelling and gene silencing. The complex consists of different subunits such as Embryonic Ectoderm Development (EED), Suppressor of Zeste 12 (SUZ12) and Enhancer of Zeste Homolog 2 (EZH2). The latter is the catalytic subunit of PRC2. The mechanism through which the PRC2 complex is recruited to chromatin remains incompletely understood. Recently, non-coding RNA has been identified as a possible player in this process^12,13^. Indeed, nearly all PRC2 subunits can bind RNA, with EZH2 having the highest affinity. EZH2 has been suggested as binding partner of numerous lncRNAs^8,14^.

The non-coding RNA cluster on chromosome 14 (14q32) is among the largest cluster of non-coding RNAs in the human genome and is well conserved among mammals. Many non-coding RNAs in this cluster have been implicated in cardiovascular disease^15^. For example, Maternally Expressed Gene 3 (MEG3) was shown to inhibit endothelial proliferation. Silencing of MEG3 improved angiogenic sprouting in vivo and in vitro^6,16,17^. Previous research has indicated a potential role for Maternally Expressed Gene 8 (MEG8) in multiple cellular processes. Terashima et al*.* showed MEG8 could contribute to epithelial-mesenchymal transition (EMT) in lung cancer cells through regulation of specific gene expression^18^. In a study by Zhang et al*.*, MEG8 was found to be downregulated in vascular smooth muscle cells (VSMC) upon ox-LDL stimulation^19^. Furthermore, loss of *Meg8* was shown to impair angiogenesis in the mouse, while overexpression of *Meg8* was shown to be protective in a rat cerebral ischemia model^20^. We therefore hypothesize that MEG8 could play a protective role in human ECs.

In this study, we report on a role for MEG8 in the regulation of angiogenic sprouting in Human Umbilical Vein Endothelial Cells (HUVECs). MEG8 was found to localize to the chromatin and shown to be involved in epigenetic regulation of genes involved in the process of angiogenesis.

## Materials and methods

### Ischemic heart disease patient (ISHD) samples

RNA was extracted from samples of left ventricular tissue obtained from 5 patients diagnosed with ischemic heart disease (ISHD) and 5 control donors (D) as described by Pham et al.^21^.

The study involving human participants was reviewed and approved by the Human Research Ethics Committee (number 2012/2814) at the University of Sydney. The patients provided informed consent to participate in the study. All procedures were in accordance with guidelines and regulations from AmsterdamUMC.Patient information can be found in supplementary table s2.

### Cell culture

HUVECs were purchased from Lonza (lots p1028 and p1032) and cultured in endothelial cell medium (ECM) supplemented with endothelial cell growth supplement (ECGS), penicillin/streptomycin (P/S) and 5% FBS (all Sciencell). HUVECs were used between passage 1 and 4 for experiments. Cells were cultured at 37 °C and 5% CO_2_. Cell numbers were determined using the Countess II Automated Cell Counter (Thermo Fisher Scientific).

### Transfection

HUVECs were transfected at 60–80% confluence with 50 nM locked nucleic acid (LNA)-GapmeR (Exiqon) or siRNA (Sigma Aldrich) using Lipofectamine RNAiMax (Thermo Fisher Scientific) in OptiMEM Glutamax (Gibco). As a control, non-targeting LNA GapmeR or siRNA was transfected. The medium was replaced with full ECM after 4 h. Sequences and catalogue numbers can be found in the supplementary table s1.

### Hypoxia

HUVEcs were transfected with Control or MEG8 GapmeR. After 24 h, cells were exposed to 1% hypoxia or normoxia for 24 h and RNA was collected. VEGFA expression was measured as a marker of hypoxia. RT-qPCR sequences and oligos can be found in supplementary table s1.

### RNA isolation and RT-qPCR

Total RNA was isolated using TRIzol (Thermo Fisher Scientific) and the Direct-zol RNA miniprep kit (Zymo Research) according to the manufacturer’s instructions. For RT-qPCR, 1000 ng of total RNA was reverse transcribed using oligo(dT) and random primers (iScript cDNA synthesis kit, BioRad) according to the manufacturer’s instructions. RT-qPCR was performed using iQ SYBR Green Supermix (BioRad) in a CFX96 or CFX384 Touch Real-Time PCR Detection System (BioRad). Gene expression analysis was done using the 2^−dCt^ method. Primer sequences are listed in the supplementary table s1.

### Spheroid assay

HUVECs were trypsinised and resuspended in ECM culture medium containing 0,6 gr/L methylcellulose (Sigma Aldrich). Cells were seeded (400 cells per well in 100 µl) in U-bottom 96 well plates (Costar) and cultured for 24 h at 37 °C and 5% CO_2_ to allow for formation of spheroids. The spheroids were collected and resuspended in FBS (Sciencell) containing 2,4 gr/L methylcellulose and mixed 1:1 with collagen I solution containing 3,77 g/L collagen I (Corning, USA), 10% M199 medium (Sigma Aldrich), 0,018 M HEPES and 0.2 M NaOH to adjust pH to 7,4. The mixture with the spheroids was allowed to polymerize for 30 min in a 24 well plate. To induce sprouting, 100 µl of ECM with or without VEGF (50 ng/mL, Preprotech) was added to the gels. Spheroids were fixed after 24 h using 10% fomaldehyde in PBS and visualized using an Olympus IX50 microscope. The cumulative sprout length per spheroid was measured using ImageJ software.

### Migration assays

A wound healing assay was performed using culture-inserts 2 well (Ibidi, 80,209). 24 h after transfection, cells were seeded in the inserts at a density of 30 000 cells per well. After another 24 h, the inserts were removed, cells were washed and cultured in ECM. The wound was imaged every hour for 8 h using the EVOS XL microscope (Thermo Fischer Scientific). Total distance was quantified using ImageJ software.

### Endothelial barrier measurements

Endothelial barrier was measured using the Electrical Cell-substrate Impedance Sensing system (ECIS, Applied Biophysics) in 8 well arrays (8W10E, Ibidi). Prior to seeding cells, the arrays were coated with 10 mM L-cysteine and 1% gelatin. Cells were seeded at a density of 100 000 cells per well. After 24 h, impedance levels stabilized, indicating a stable barrier. An electrical pulse (5 V, 6000 Hz) was applied to remove a section of the monolayer and create a wound. Impedance measurements were continued until a plateau was reached again.

### Proliferation assay

Cell proliferation was measured using a 5-ethynyl-2’-deoxyuridine (EdU) incorporation assay (Click-iT EdU cell proliferation kit, Thermo Fisher Scientific). 20 h after transfection, cells were seeded in 8 well µ-slides (Ibidi) at a density of 30 000 cells per well and left to reattach for 4 h. EdU (final concentration 10 µM) was added to each well and fixation and staining was done after another 24 h. Cells were imaged using the Axio Observer Z1.0 microscope (Zeiss). Total cell number and EdU positive cells were counted.

In addition, cell numbers were counted over time. HUVECs were seeded at equal densities and counted 24-, 48- and 72 h after transfection using the Countess II Automated Cell Counter (Thermo Fisher Scientific).

### SCRINSHOT RNA FISH

SCRINSHOT RNA FISH was described by Sountoulidis et al.^22^. Cells were seeded in 12-well removable slides (81201, Ibidi) coated with 1% gelatin. Cells were fixed after 24 h with 4% paraformaldehyde (PFA). Permeabilization was done using 0.1 M HCl. Blocking was done using blocking solution (0.1 M oligo-dT (Sigma Aldrich), Ampligase buffer (Lucigen), 0.05 M KCl, 20% formamide (Sigma Aldrich), 0.2 μg/μl BSA (NEB), 1 U/μl Ribolock (Thermo Fisher Scientific), 0.2 μg/μl tRNA’s (Sigma Aldrich)) for 30 min. Padlock probes were incubated for 15 min at 55 °C and 120 min at 45 °C. Slides were washed with 10% formamide in 2 × SSC buffer (Sigma Aldrich) and ligated with SplintR Ligase (NEB) for 16–24 h at 25 °C. Rolling circle amplification was performed using Φ29 polymerase (Lucigen) and the following primer: TAAATAGACGCAGTCAGT ∗ A ∗ A. The * indicates thiophosphate-modified bounds to inhibit the 3–5 exonuclease activity of Φ29 polymerase. The amplified products were fixated with 4% PFA and detection oligo’s were incubated at RT for 60 min. Oligo sequences can be found in the supplementary table s1. Coverslips were mounted with SlowFadeTM Gold Antifade mounting medium (Thermo Fisher Scientific). Cells were imaged using the Nikon A1R confocal microscope.

### RNA sequencing

HUVECs were transfected with Control or MEG8 GapmeRs. Total RNA was isolated after 48 h using TRIzol (Thermo Fisher Scientific) and the Direct-zol RNA miniprep kit (Zymo Research) according to the manufacturer’s instructions. Total RNA and library integrity were verified on LabChip Gx Touch 24 (Perkin Elmer). 1 µg of total RNA was used as input for SMARTer Stranded Total RNA Sample Prep Kit—HI Mammalian (Clontech). Sequencing was performed on the NextSeq500 instrument (Illumina) using v2 chemistry and 1 × 75 bp single end setup. The resulting raw reads were assessed for quality, adapter content and duplication rates with FastQC (Available online at: http://www.bioinformatics.babraham.ac.uk/projects/fastqc). Trimmomatic version 0.39 was employed to trim reads after a quality drop below a mean of Q20 in a window of 10 nucleotides^23^. Only reads of at least 15 nucleotides were cleared for subsequent analyses. Trimmed and filtered reads were aligned versus the Ensembl human genome version hg38 (GRCh38) using STAR 2.6.1d with the parameter “–outFilterMismatchNoverLmax 0.1” to increase the maximum ratio of mismatches to mapped length to 10%^24^. The number of reads aligning to genes was counted with featureCounts 1.6.5 from the Subread package^25^. Only reads mapping at least partially inside exons were admitted and aggregated per gene. Reads overlapping multiple genes or aligning to multiple regions were excluded. Differentially expressed genes were identified using DESeq2 version 1.18.1^26^. Only genes with a minimum fold change of +  −  1.5 (log2 +  − 0.585), a maximum Benjamini–Hochberg corrected p-value of 0.05, and a minimum combined mean of 5 reads were deemed to be significantly differentially expressed. The Ensemble annotation was enriched with UniProt data (release 24.03.2017) based on Ensembl gene identifiers (Activities at the Universal Protein Resource (UniProt)). Sequencing data was deposited under GEO accession number GSE186616.

### Western blot

HUVECs were lysed 48 h after transfection in radioimmunoprecipitation assay (RIPA) buffer (Sigma Aldrich) with protease and phosphatase inhibitors (Halt inhibitor cocktail, Thermo Fisher Scientific). Lysates were centrifuged at 20.000 g for 10 min and protein concentration was measured using Pierce BCA protein assay kit (Thermo Fisher Scientific). Equal amounts (10 µg) of protein were separated on SDS-PAGE gels and blotted on nitrocellulose membranes. Membranes were blocked in block buffer (TBST + 5% BSA + 0.05% sodium azide) and incubated overnight at 4 °C with primary antibodies diluted in block buffer. Secondary antibodies (Dako) were incubated for 2 h at room temperature. Bands were visualized using enhanced chemiluminescence (ECL, Amersham/GE-healthcare) on the AI600 (Amersham/GE-healthcare). Original full length blots can be found in supplementary Fig. 4. Antibodies and dilutions can be found in supplemental table s1.

### Cellular fractionation

RNA was isolated from untransfected HUVECs in cytoplasmic, nucleoplasm and chromatin fractions. Cells were collected and washed in cold PBS. Cells were lysed in cytoplasmic lysis buffer (10 mM Tris (pH 7.5), 150 mM NaCl, 0.15% NP-40), layered on a sucrose buffer (10 mM Tris (pH 7.5), 150 mM NaCl, 24% (w/v) sucrose) and centrifuged for at 4 °C for 10 min at 16.000 × g. The supernatant (cytoplasmic fraction) was added to TRIzol LS (Thermo Fisher Scientific) for RNA isolation and the pellet was resuspended in glycerol buffer (20 mM Tris (pH 7.9), 75 mM NaCl, 0.5 mM EDTA, 0.85 mM DTT, 50% glycerol). Nuclei lysis buffer (10 mM HEPES (pH 7.6), 7.5 mM MgCl2, 0.2 mM EDTA, 0.3 M NaCl, 1 M urea, 1 mM DTT, 1% NP-40) was added, incubated in ice and centrifuged for 2 min at 4 °C and 16.000 × g. The supernatant (nucleoplasm fraction was) resuspended in TRIzol LS. The pellet (chromatin fraction) was resuspended in cold PBS and vortexed to release the RNA. TRIzol LS was added and RNA was isolated using the Direct-zol RNA miniprep kit (Zymo Research). Equal volumes were used for subsequent cDNA synthesis to ensure comparison of equal cell equivalents.

### Chromatin immunoprecipitation (ChIP)

ChIP was performed using the EZ Magna ChIP G kit (14–409 Millipore). One confluent 15 cm dish of HUVECs was used per condition. MEG8 and control GapmeRs were transfected 48 h prior to starting the ChIP protocol. DNA extraction was done by adding an equal volume phenol/chlorophorm/isomylalcohol (P2069-100ML, Sigma Aldrich). Phase separation was performed by centrifugation (12 000 × g, 10 min). 0.1X volume sodium acetate (3 M), 1 µl glycogen and 0.7X volume isopropanol was added and DNA was precipitated at − 80 °C. Washing was done using 75% ethanol and the pellet was resuspended in Ultrapure water (Invitrogen). All samples and 1% input were analysed using RT-qPCR according to the instructions from the kit. Antibodies were used at a concentration of 1 µg per condition. Antibodies and primers can be found in the supplementary table s1.

### RNA immunoprecipitation

For RNA immunoprecipitation (RIP), 5 µg or equivalent volume antibody was coupled to 50 µl Dynabeads protein G beads (10003D, Thermo Fisher) overnight at 4 °C under rotation. One confluent 15 cm dish of HUVECs was used per condition. HUVECs were crosslinked with 50 mJ/cm2 UV light, washed in PBS and lysed with lysis buffer (50 mM Tris–HCl pH8, 150 mM NaCl, 1 mM EDTA, 0.5% NP-40 and protease inhibitors). The supernatant was cleared by centrifugation (10 000 × g for 10 min at 4 °C) and the supernatant was diluted in 1 mL lysis buffer without NP-40. Protein G beads were washed 3 × in binding buffer (50 mM Tris/HCl pH8, 150 mM NaCl, 1 mM EDTA, 0.05% NP-40) and lysate was incubated with the beads for 4 h at 4 °C under rotation. Beads were washed in binding buffer and treated with proteinase K (6.4 U per tube, NEB P8107S) in proteinase K buffer (200 mM Tris HCl pH8, 25 mM EDTA, 300 mM NaCl, 2% SDS) for 30 min at 50 °C. An equal volume of phenol/chlorophorm/isomylalcohol (Roth) was added for RNA extraction. Phase separation was done by centrifugation (20 000 × g, 10 min). 2,5 X volume ethanol was added and RNA was precipitated overnight at − 20 °C. Washing was done using 70% ethanol and pellet was resuspended in MilliQ. cDNA was transcribed using the iScript kit. MEG8, MALAT1 and GAPDH enrichment was measured by RT-qPCR.

### Statistical analysis

Data is presented as mean ± standard error of the mean (SEM). GraphPad Prism 8 was used for the analysis. When comparing 2 groups, a student’s t-test or Mann–Whitney test was performed. When comparing more than 2 groups, analysis of variance (ANOVA) was performed including Holm- Sidak correction for multiple testing. A p-value < 0.05 was considered significant.

## Results

### MEG8 is involved in angiogenic sprouting and cell proliferation but not migration

**Figure 1:**
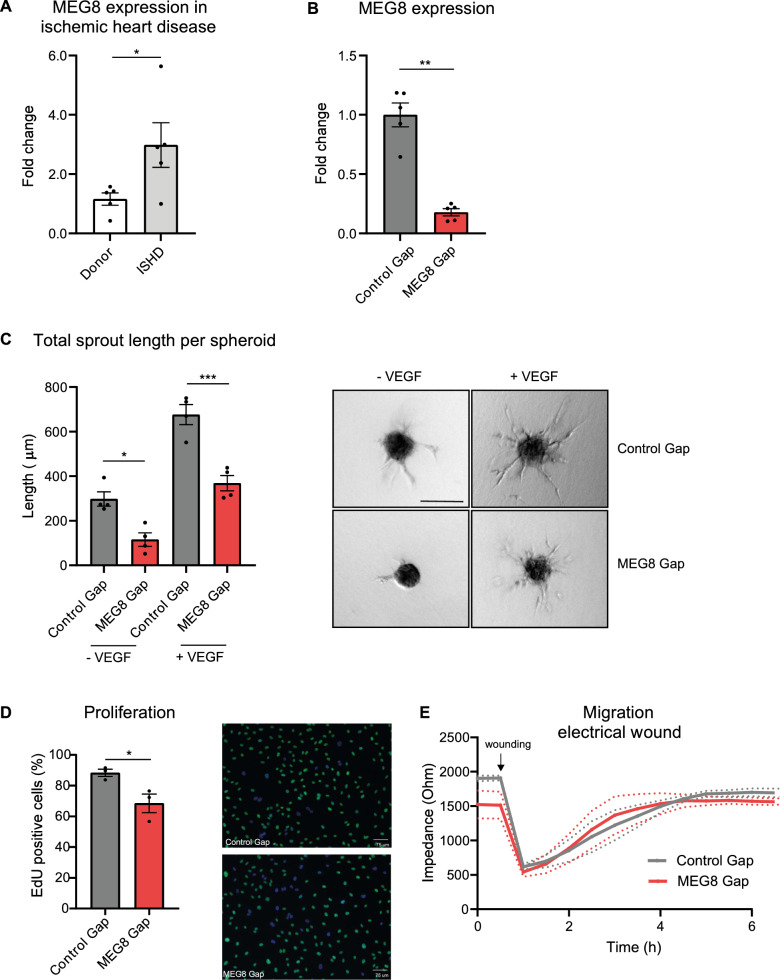
Knockdown of MEG8 results in impaired EC sprouting and proliferation. (**A**) MEG8 expression was measured by RT-qPCR in ischemic heart disease patients, relative to donors. Expression is normalized to RPLP0. (**B**)-(**E**): HUVECs were transfected with MEG8 or control GapmeR and (**B**) expression levels were measured after 48 h by RT-qPCR. Expression is relative to RPLP0 (**C**) EC spheroids were embedded in collagen gels 24 h after transfection and stimulated with VEGF. Fixation was done after 24 h VEGF stimulation. Cumulative sprout length was determined by measuring the distance from the base of the spheroid to the tip cell. Discontinuous sprouts were excluded. For 4 independent experiments, 7–12 spheroids were scored in each experiment. Scale bar indicates 75 µm. (**D**) Proliferation was measured by EdU incorporation between 24–48 h after transfection. The percentage of proliferating cells is shown. Scale bar indicates 75 µm. (**E**) The effect of MEG8 knockdown on migration was assessed by ECIS. Cells were seeded in ECIS slides 24 h after transfection. After 24 h a monolayer had been established, and an electrical pulse was applied to create a cell free area. Electrical impedance was measured until a plateau was reached. Data are presented as mean ± SEM. A comparison of two groups was estimated by unpaired t-test. Multiple groups were analysed using one-way ANOVA. Significance was indicated as: *p < 0.05, **p < 0.01, ***p < 0.001.

Angiogenesis is crucial to restore blood flow after ischemic disease. To better understand how this process is regulated, we obtained samples from the left ventricle of ISHD patients. An important role for the 14q32 cluster had previously been described^27^. MEG3 is a negative regulator of angiogenesis^16,17^. The role of another lncRNA in this cluster, MEG8, was less understood. We sought to further characterize MEG8 function. MEG8 expression was upregulated threefold in ISHD patients compared to left ventricle tissue from control donors with no history of heart disease (Fig. 1A). This suggests a potentially interesting role for MEG8 in cardiovascular disease. Coding Potential Assessing Tool (CPAT) analysis indicated a low coding potential for MEG8, similar to known lncRNAs such as MALAT1 and XIST (Supplementary Fig. 1A). To understand the function of MEG8 in the endothelium, we silenced MEG8 expression in HUVECs using LNA-GapmeRs. Total MEG8 levels were reduced by around 80% compared to a non-targeting control (Fig. 1B). Silencing of MEG8 resulted in a 60% reduction in angiogenic sprouting, as assessed by an in vitro sprouting angiogenesis assay (Fig. 1C). Stimulation of sprouting with VEGF resulted in increased sprout length in all conditions, however, sprouts in the MEG8 silenced conditions were 44% reduced compared to their respective control (Fig. 1C). This finding was validated using siRNA mediated knockdown of MEG8 (Supplemental Fig. 2A). To exclude the possibility that MEG8 regulates VEGF expression, which could contribute to impaired sprouting, we assessed VEGF mRNA and protein levels. We observed no changes in VEGF mRNA levels after loss of MEG8 (Supplemental Fig. 2C). We observed a reduction in VEGF protein (Supplemental Fig. 2D). Adding exogenous VEGF, however, did not restore sprouting in MEG8 silenced cells (Fig. 1C, supplemental Fig. 2A). Multiple processes are involved in forming new vessels, such as proliferation and migration. To better understand how MEG8 regulates sprouting, both migration and proliferation were assessed in MEG8 silenced HUVECs. Proliferation, as measured by an EdU incorporation assay, was reduced by approximately 20% after both GapmeR and siRNA mediated loss of MEG8 (Fig. 1D, supplemental Fig. 2B). A cell wounding protocol in the ECIS system was used to investigate migration. In this setup, a cell free area is created by lethal electroporation using a high frequency current. Surrounding cells migrate to re-establish a monolayer, as indicated by increased impedance. The slope of the curve was similar in both conditions, indicating cells migrated at a similar speed (Fig. 1E, supplemental Fig. 1B). These findings were validated using a scratch healing assay (Supplemental Fig. 1C). In both methods, migration was assayed within 8 h to ensure the observed effects are not due to proliferation. Both assays show no change in EC migration capacity after loss of MEG8. Taken together, these results suggest MEG8 regulates angiogenic sprouting, possibly through the regulation of proliferation.

**Figure 2:**
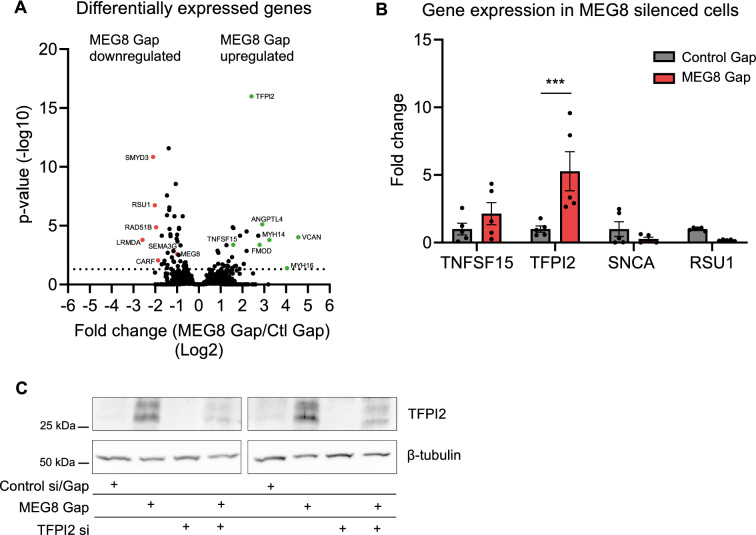
TFPI2 induction following loss of MEG8. (**A**) Changes in gene expression after GapmeR-mediated silencing of MEG8 were analysed by RNA sequencing. RNA was collected 48 h after transfection. (**B**) A subset of genes related to angiogenesis was validated by RT-qPCR. TFPI2 was significantly induced. Expression is relative to RPLP0. (**C**) TFPI2 protein levels were determined using Western blot. Cell lysates were collected 48 h after transfection. β-tubulin was used as a loading control. Images were cropped for clarity. Data is presented as mean ± SEM. Groups were analysed using one-way ANOVA. Significance was indicated as: *p < 0.05, **p < 0.01, ***p < 0.001.

### Differential expression of angiogenesis related genes after loss of MEG8

Regulation of transcription is a key function for many lncRNAs^9^. To determine whether MEG8 affects gene expression, RNA sequencing was performed in HUVECs after treatment with control or MEG8 GapmeR (Fig. 2A, supplemental Fig. 2E). We observed 76 genes which were differentially expressed after loss of MEG8, suggesting MEG8 does not greatly affect global gene transcription but rather a specific set of genes (Supplemental Fig. 2E). Interestingly, a number of genes that were differentially expressed had been shown previously to be involved in the regulation of angiogenesis. Tumor necrosis factor superfamily-15 (TNFSF15) is expressed mainly in ECs where it inhibits capillary formation and has been shown to induce apoptosis in proliferating ECs^28^. Angiopoietin-like 4 (ANGPTL4) has been shown to act as both a pro- and anti-angiogenic factor^29,30^. Tissue Factor Pathway Inhibitor 2 (TFPI2) is a serine protease inhibitor which has been reported to inhibit trypsin, factor XIa as well as matrix metalloproteinases (MMPs). TFPI2 likely plays a role in the coagulation cascade and extracellular matrix remodelling. Interestingly, studies have suggested an inhibitory role for TFPI2 in angiogenesis^31–33^. Several genes were validated by RT-qPCR (Fig. 2B). We could confirm that loss of MEG8 resulted in a fivefold upregulation of Tissue Factor Pathway Inhibitor 2 (TFPI2) mRNA. There was also an upregulation of TFPI2 protein after loss of MEG8 (Fig. 2C). In concord, TFPI2 expression tends to be reduced in ISHD patients compared to donors (Supplemental Fig. 2F). In addition, we exposed HUVECs to 24 h of 1% hypoxia to mimic ischemic conditions. We observed an induction of TFPI2 after knockdown of MEG8 in both hypoxic and normoxic conditions (Supplemental Fig. 2G). We conclude that regulation of TFPI2 by MEG8 occurs both under basal conditions in the endothelium as well as during ischemia. These results suggest MEG8 regulates expression of a number of genes related to angiogenesis.

### Reduced histone 3 lysine 27 trimethylation of the TFPI2 promotor after loss of MEG8

**Figure 3:**
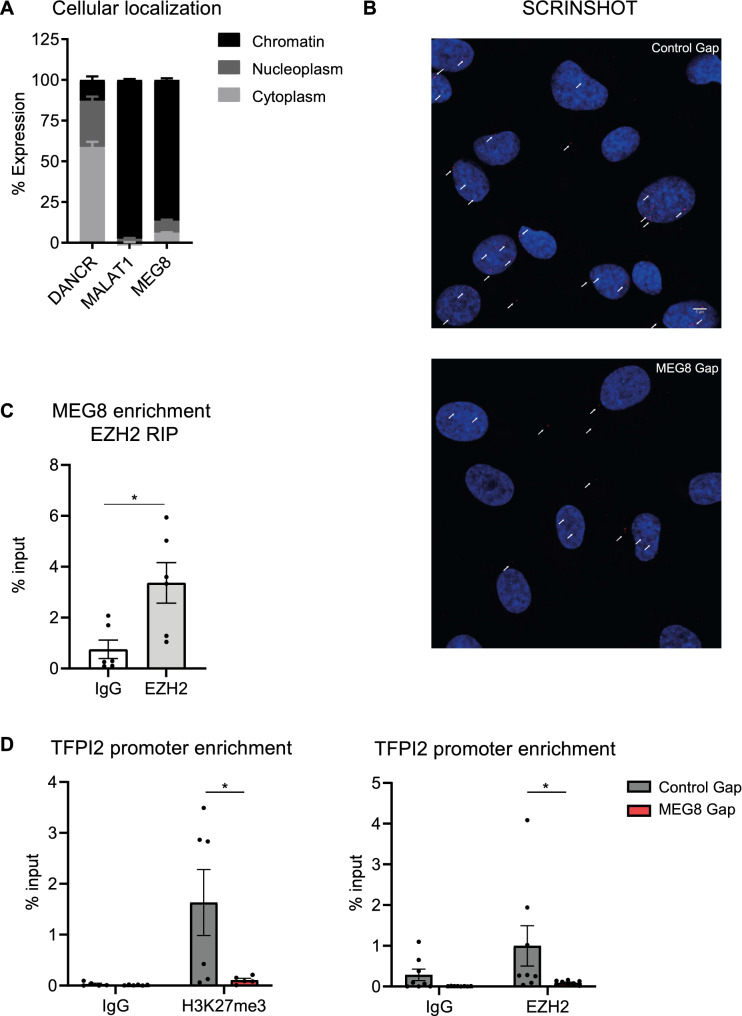
Reduced H3K27 trimethylation of the TFPI2 promotor after loss of MEG8. (**A**) RNA was extracted from the chromatin, nucleoplasm and cytoplasm of HUVECs. RT-qPCR was used to analyse MEG8 localization. DANCR and MALAT1 were used as a control for cytoplasm or nuclear transcripts, respectively. (**B**) HUVECs were treated with MEG8 or control GapmeR for 48 h. Subcellular localization of MEG8 (in red) was analysed by SCRINSHOT RNA FISH. Nuclei and membrane were immunostained with DAPI (blue). Scale bar indicates 5 µm. (**C**) MEG8 binding to EZH2 was analysed in HUVECs by RT-qPCR following immunoprecipitation. Non-targeting IgG was used as a control. Enrichment was quantified relative to input. Data are presented as mean ± SEM. Analysis was done using unpaired *t* test. (**D**) Chromatin immunoprecipitation assay of H3K27me3 binding and EZH2 occupancy at the TFPI2 promoter. HUVECs were treated with control or MEG8 GapmeR 48 h prior to fixation. Enrichment was quantified relative to input. IgG was used as a negative control. Data are presented as mean ± SEM. H3K27me3 ChIP was analysed using one-way ANOVA and EZH2 ChIP was analysed using a Friedman test. Significance was indicated as: *p < 0.05, **p < 0.01, ***p < 0.001.

Cellular fractionation assays have shown MEG8 to be mainly located in the nucleus (Fig. 3A). MEG8 was found to specifically localize to the chromatin, as compared to the cytoplasmic lncRNA DANCR^34^ and nuclear retained lncRNA MALAT1^35^. We confirmed this by SCRINSHOT RNA FISH (Fig. 3B, Supplemental Fig. 3A). These results suggest MEG8 could be involved in epigenetic regulation of gene expression. In study by Terashima et al.^18^, MEG8 was shown to mediate EZH2 recruitment to specific promotor sites and affect histone 3 (H3) methylation in pancreatic cancer cells. EZH2, part of the PRC2 complex, is well known to induce chromatin remodelling and gene silencing^12^. EZH2 immunoprecipitation and RT-qPCR confirmed interaction between MEG8 and EZH2 in our EC model (Fig. 3C). Previous research showed that EZH2 can silence TFPI2 expression in glioblastoma^36^. To further address whether MEG8 regulates TFPI2 expression through interacting with EZH2 in the endothelium, Chromatin Immunoprecipitation (ChIP) assays were performed (Fig. 3D). Since EZH2 is known to trimethylate H3K27 to induce silencing of transcription, this modification was assessed as a measure of EZH2 recruitment^8,14^. Indeed, after silencing of MEG8, H3K27 trimethylation of the TFPI2 promoter was significantly reduced by an average of 80% (Fig. 3D). In addition, EZH2 enrichment to the TFPI2 promoter is reduced after MEG8 silencing (Fig. 3D). Taken together, these results suggest there is less H3K27me3-mediated inhibition of TFPI2 expression after loss of MEG8.

### MEG8 is required for angiogenesis via reduction of TFPI2 expression

**Figure 4:**
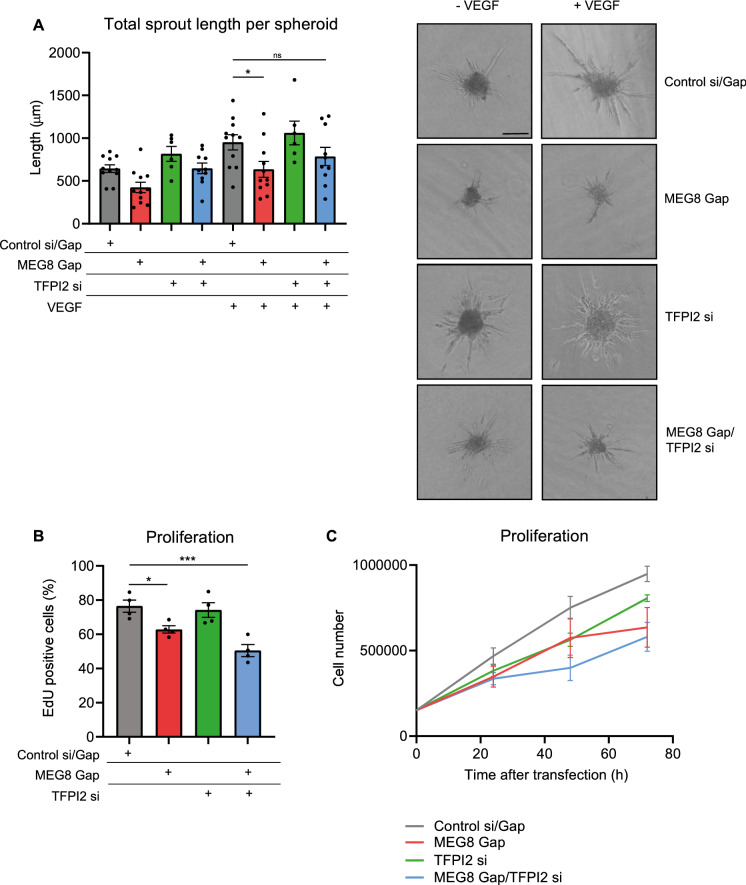
TFPI2 silencing rescues impaired sprouting after loss of MEG8. MEG8 and TFPI were knocked down by GapmeR and siRNA, respectively. (**A**) EC spheroids were embedded in collagen gels 24 h after transfection and stimulated with VEGF. Spheroid were fixed after 24 h VEGF stimulation. Cumulative sprout length was determined by measuring the distance from the base of the spheroid to the tip cell. Discontinuous sprouts were excluded. For each independent experiment 7–15 spheroids were scored. 10 independent experiments were performed. Scale bar indicates 100 µm. Multiple groups were analysed using one-way ANOVA. (**B**) Proliferation was measured by EdU incorporation between 24–48 h after transfection. The percentage of EdU-positive cells is shown. Multiple groups were analysed using one-way ANOVA (**C**) In addition, cell numbers were counted over time. HUVECs were seeded at equal densities and counted 24-, 48- and 72 h after transfection. Data are presented as mean ± SEM. Multiple groups were analysed using one-way ANOVA. Significance was indicated as: *p < 0.05, **p < 0.01, ***p < 0.001.

We hypothesized that by inhibiting TFPI2, which is induced after MEG8 silencing, there could be a rescue of angiogenic sprouting. To achieve this, we silenced TFPI2 using siRNA-mediated knockdown. We were able to prevent TFPI2 induction following MEG8 knockdown by transfection of TFPI2 siRNA (Fig. 2C). We observed no change in MEG8 expression following TFPI2 knockdown (Supplemental Fig. 3B). As expected, silencing of MEG8 resulted in disturbed sprouting (Fig. 4A). Knockdown of TFPI2 resulted in increased sprout length, although this was not statistically significant. Silencing of both MEG8 and TFPI2 partially restored sprouting capacity compared to MEG8 silencing alone. This effect was most pronounced in VEGF stimulated cells. These results suggest MEG8 contributes to regulation of angiogenic sprouting, at least in part through the regulation of TFPI2 expression. Proliferation assays had indicated that MEG8 silencing resulted in reduced proliferation, thought to contribute to impaired sprouting. We therefore investigated how TFPI2 affects EC proliferation. When MEG8 was silenced, proliferation was reduced, as measured by both EdU incorporation (Fig. 4B) and cell counting (Fig. 4C). Additional silencing of TFPI2 did not rescue proliferation levels. Also, TFPI2 silencing alone did not greatly affect proliferation. This finding would suggest that TFPI2 regulates angiogenesis through a mechanism other than proliferation.

## Discussion

This study identifies a role for lncRNA MEG8 in the regulation of angiogenesis. Loss of MEG8 in ECs is accompanied by impaired angiogenic sprouting and proliferation. Furthermore, TFPI2 expression was induced following loss of MEG8. Mechanistically, MEG8 was found to interact with EZH2, which is part of the PRC2 complex. Accordingly, silencing of MEG8 resulted in a reduction of repressive H3K27me3 mark at the TFPI2 promoter. Silencing of TFPI2 rescued sprouting capacity of MEG8 deficient cells.

MEG8 is found in the 14q32 cluster in humans and 12F1 in mice. Recently, Sui et al*.* showed that downregulation of *Meg8*, also known as *Rian* in mice, inhibited cell viability and angiogenesis in mouse brain microvascular ECs, in accordance with our findings in a HUVEC model^20^. Voellenkle et al*.* showed an upregulation of MEG8 expression in HUVECs exposed to hypoxia^37^. We observed an induction of MEG8 expression in ISHD patients (Fig. 1A). Taken together, these results point to a possible protective role for MEG8 in ischemia. We hypothesize that MEG8 is upregulated during ischemia to contribute to cell survival and angiogenesis. A study by Zhang et al*.* identified a role for MEG8 in VSMC proliferation and migration through targeting PPARα. Contrary to findings in the endothelium, inhibition of MEG8 improved proliferation following PPARα. Enhanced expression of MEG8 was found to repress proliferation and migration of VSMCs^19^. These results hint to a cell-specific function of MEG8.

Our results show a reduction in sprouting and proliferation (Fig. 1C,D), but not migration (Fig. 1E) after loss of MEG8. Extension of the sprouts, mediated by stalk cell proliferation, is therefore more likely to be disturbed when MEG8 is silenced. Sui et al*.* show reduced *Vegf* expression following loss of *Meg8* in a mouse model which would result in impaired angiogenesis^20^. This mechanism does not seem to play a major role in our human angiogenesis model since stimulation of spheroids with exogenous VEGF did not rescue sprouting following MEG8 knockdown. In addition, we observed no change in VEGF mRNA expression after loss of MEG8 (Supplementary Fig. 2C). VEGF protein levels were reduced following loss of MEG8 (Supplementary Fig. 2D). However, since VEGF expression was not regulated at the transcriptional level, it is possible that this effect is secondary to other effects observed after loss of MEG8 (Fig. 4A). This would suggest that, in human ECs, MEG8 regulates angiogenesis at least in part independent of VEGF expression. Interestingly, MEG3, a lncRNA upstream of MEG8 in the 14q32 cluster has been shown to play a role in angiogenesis as well. Contrary to *Meg8*, inhibition of *Meg3* was shown to induce angiogenesis in mice^16,38^. Mechanistically, *Meg3* was shown to negatively regulate the Notch pathway. Chen et al*.* showed differential expression of Notch genes (*Notch2, Notch3, Hes1*) in murine hepatic stellate cells after *Meg8* knockdown^39^. We did not observe any change in Notch gene expression following loss of MEG8 in the human endothelium (Fig. 2A). These findings would suggest these two lncRNAs from the same cluster function in different cellular pathways.

MEG8 was found to localize to the chromatin (Fig. 3A), which suggested that MEG8 could be involved in epigenetic regulation of transcription, as has been shown previously for many nuclear lncRNAs^9^. RNA sequencing data suggested that MEG8 regulates expression of specific genes such as TFPI2 rather than global gene transcription (Fig. 2A,B). TFPI2 was selected as an interesting target since it has been suggested to play an inhibitory role in angiogenesis. We observed a trend towards reduced TFPI2 expression in ISHD patients compared to control (Supplementary Fig. 2F). The limited number of samples is a likely reason we did not observe a statistically significant difference. Overexpression of TFPI2 was found to inhibit capillary formation in vitro^31–33^. Modulation of angiogenic growth factor levels has been proposed as a mechanism for this anti-angiogenic effect^32^. Interestingly, TFPI2 was found to be repressed in invasive cancers^40,41^.

We then sought to elucidate the underlying mechanism. MEG8 was found to interact with EZH2 (Fig. 3C) in primary ECs and there was a reduction in the repressive H3K27me3 mark at the TFPI2 promoter after MEG8 silencing (Fig. 3D). These results suggest MEG8 mediates EZH2 recruitment and subsequent H3K27 trimethylation of specific genomic regions. We hypothesized that inhibition of TFPI2 induction by MEG8 silencing could rescue the effect of MEG8 silencing. Indeed, silencing of TFPI2 rescued sprouting following loss of MEG8 (Fig. 4A). These results suggest that MEG8 regulates angiogenic sprouting at least in part through regulation of TFPI2 expression. In addition, we measured the rate of proliferation, as this contributes to sprout extension. Knockdown of MEG8 resulted in impaired sprouting, we therefore asked whether TFPI2 was involved in regulation of proliferation as well. In previous studies, TFPI2 was shown to be induced by VEGF in the endothelium. Modulation of growth factor levels was proposed as a mechanism for the anti-angiogenic effects of TFPI2^31^. TFPI2 was observed in turn to inhibit VEGF induced proliferation, suggesting a negative feedback loop to control cellular turnover^31,42^. This mechanism appears less likely to play a role in our angiogenesis model since stimulation of spheroids with VEGF did not rescue sprouting. In our model, additional silencing of TFPI2 did not rescue proliferation rates in HUVECs after silencing of MEG8 (Fig. 4B,C). This result suggests that the effect of TFPI2 on sprouting is independent of proliferation. The degradation of the basement membrane by MMPs is a crucial first step in the sprouting phase of angiogenesis^43,44^. TFPI2 is deposited in the extracellular matrix, inhibits a range of proteases such as MMPs and plasmin and protects the matrix from degradation. TFPI2 was found to reduce activity of MMP-1, -2, -9 and -13 and weakly inhibits coagulation proteins^45^. Overexpression of TFPI2 prevented tumour invasion and metastasis in pancreatic cancer^46^. We hypothesize that MEG8 could play a role in regulating TFPI2 protein levels and thereby its deposition in the extracellular matrix and inhibition of MMPs. Remodelling of the extracellular matrix is potentially mediated through the MEG8/TFPI2 axis, and thereby contributes to angiogenesis.

Taken together, our study shows that MEG8 is regulated in ISHD and controls angiogenesis via epigenetic regulation of TFPI2 expression. Our study further highlights the MEG8/TFPI2 axis as potential therapeutic approach to improve angiogenesis in ischemia.

## Supplementary Information

Supplementary Information.
