# Supplementary material for: MEG8 regulates Tissue Factor Pathway Inhibitor 2 (TFPI2) expression in the endothelium
Source: Sci Rep. 2022 Jan 17;12:843. doi: 10.1038/s41598-022-04812-z (PMC8763909; doi:10.1038/s41598-022-04812-z)
Supplement: Supplementary file 1 — Supplementary Information. [file 41598_2022_4812_MOESM1_ESM.pdf]

# MEG8 regulates Tissue Factor Pathway Inhibitor 2 (TFPI2) expression in the endothelium

Veerle Kremer[1,2], Diewertje I. Bink[1], Laura Stanicek[1,5], Eva van Ingen[3], Theresa Gimbel[5,6], Sarah Hilderink[1], Stefan Günther[7,8], Anne Yaël Nossent[3,4], Reinier A. Boon[1,5,6,*]

1 Department of Physiology, Amsterdam Cardiovascular Sciences, VU Medical Center, Amsterdam UMC, The Netherlands
2 Department of Medical Chemistry, Academic Medical Center, Amsterdam UMC, The Netherlands
3 Department of Surgery, The Netherlands Einthoven Laboratory for Experimental Vascular Medicine, Leiden University Medical Center, The Netherlands
4 Departments of Laboratory Medicine and Internal Medicine II, Medical University of Vienna, Austria
5 Institute of Cardiovascular Regeneration, Goethe University, Frankfurt am Main, Germany
6 German Centre for Cardiovascular Research DZHK, Partner site Frankfurt Rhein/Main, Frankfurt am Main, Germany
7 DZHK German Centre for Cardiovascular Research, Partner Site Rhine-Main, Bad Nauheim, Germany
8 Max Planck Institute for Heart and Lung Research, Bioinformatics and Deep Sequencing Platform, Bad Nauheim, Germany
* Corresponding author: r.a.boon@amsterdamumc.nl. Amsterdam UMC, De Boelelaan 1108, 1081 HZ Amsterdam.

**Supplementary figure 1**

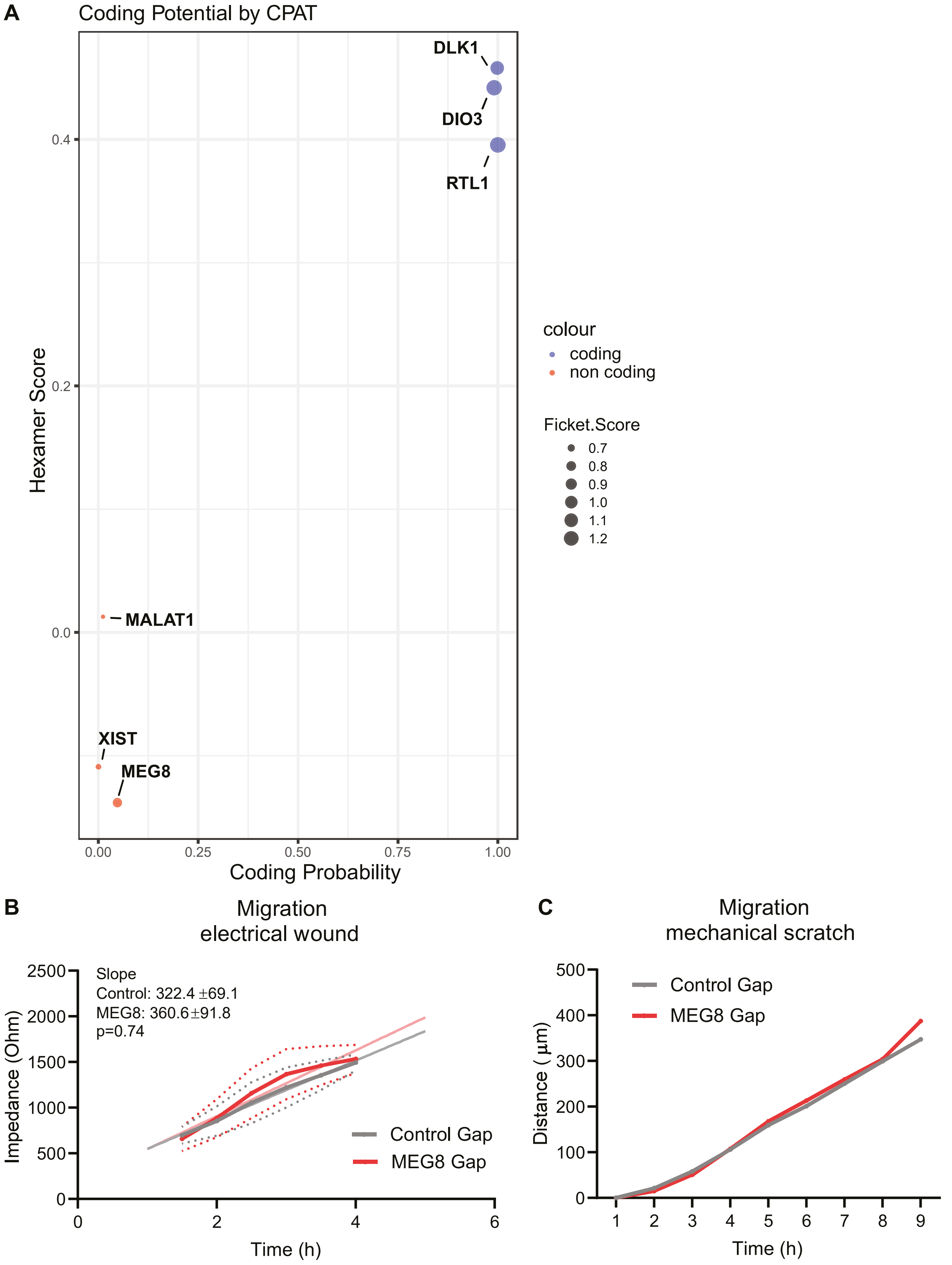

## Supplementary figure 2

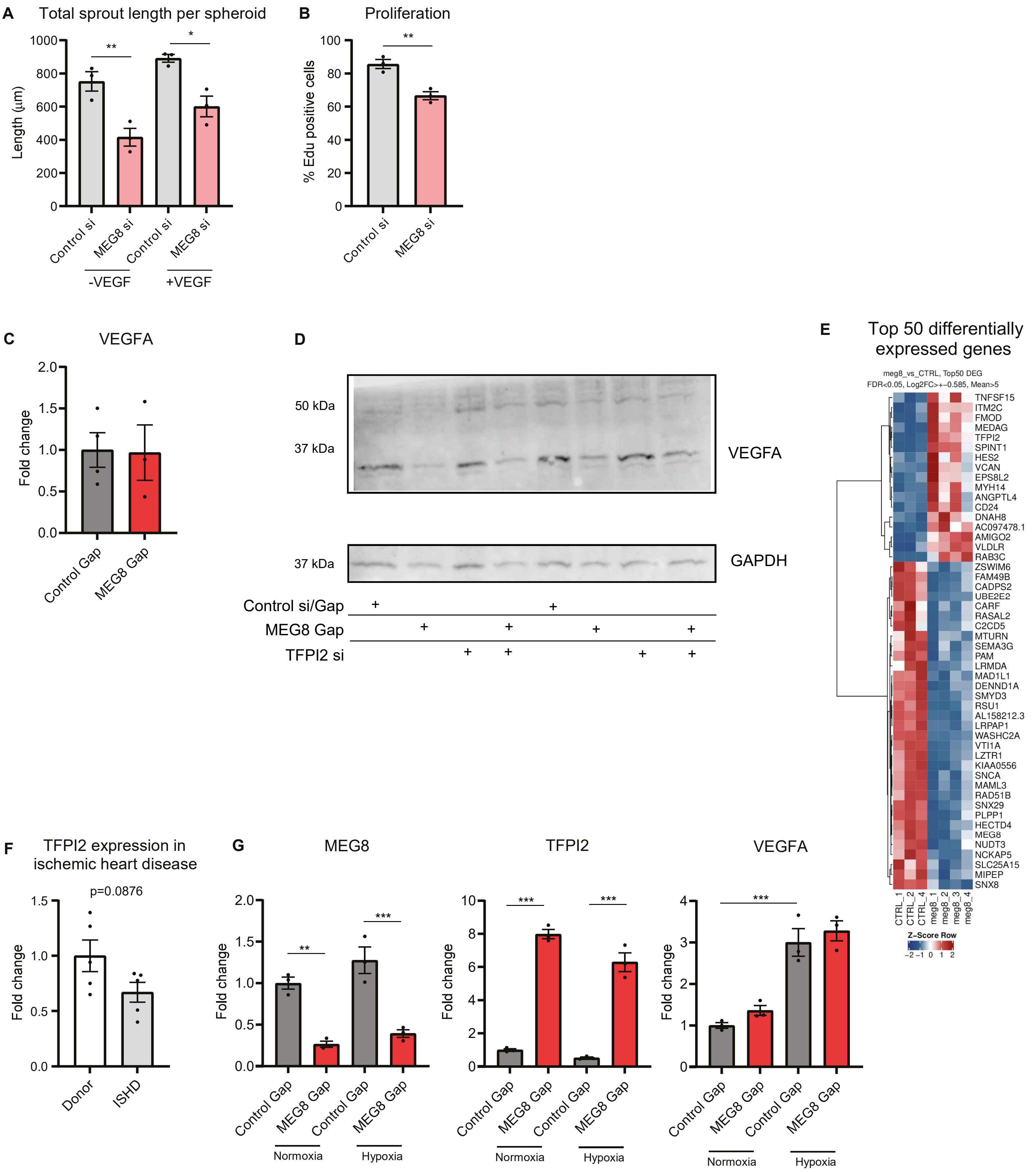

**Supplementary figure 3**

A

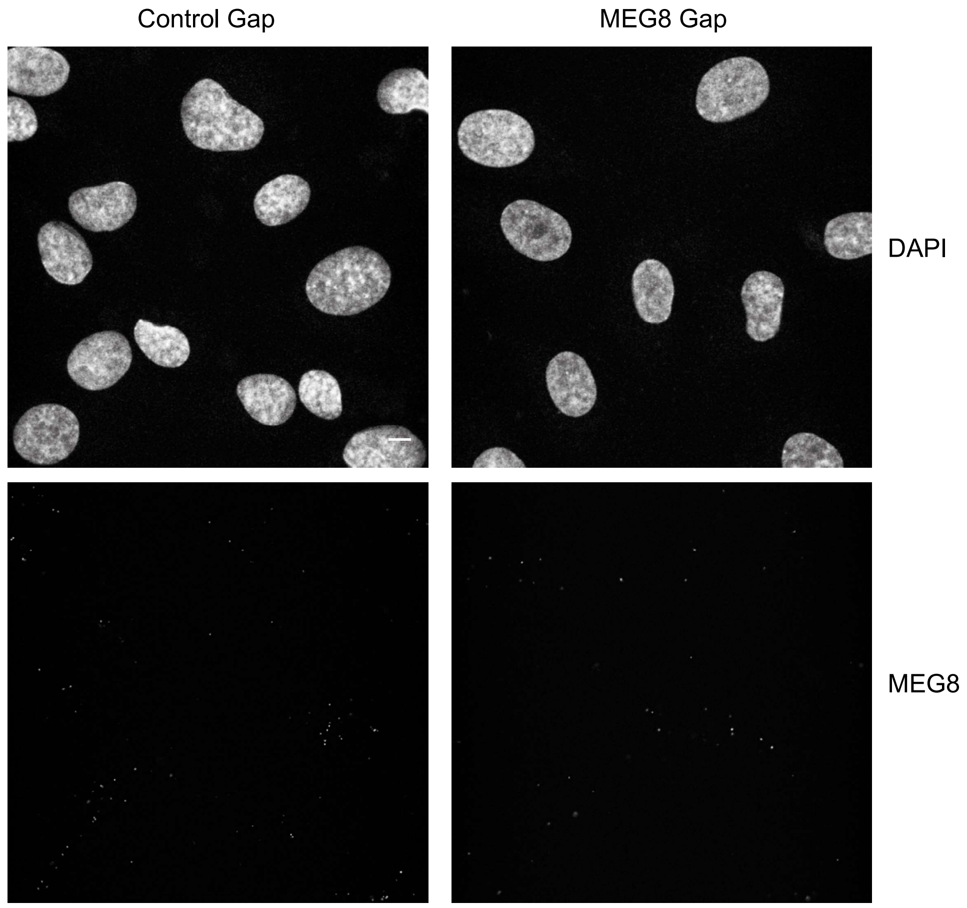


B

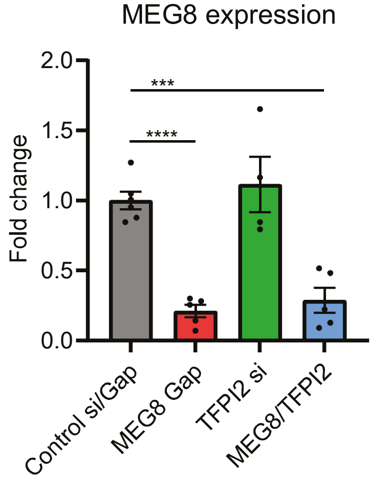

## Supplementary table s1

**Human primers for RT-qPCR**

| Gene | Forward | Reverse | Manufacturer | Source |
|---|---|---|---|---|
| MEG8 | GAGGACTTGGAGAGGTTAGTGAC | ACCTGAGTTGGAATCCTGGG | Sigma Aldrich | |
| MEG8-2 | CTCTGTGAATCAGGAGAGAAGAGA | TTCACCTTGGGGAAATGACCA | | PMID:31711641 |
| RPLP0 (p0) | TCGACAATGGCAGCATCTAC | ATCCGTCTCCACAGACAAGG | | |
| GAPDH | ATGGAAATCCCATCACCATCTT | CGCCCCACTTGATTTTGG | | |
| TFPI2 | GGGCCCTACTTCTCCGTTAC | CACACTGGTCGTCCACACTC | | |
| TFPI2 promoter | GGAATTCCCCGCCAAGTT | CCGTCTGGACTACAGGAGAAAGTT | | PMID: 23703216 |
| ACTB promoter | TCCCCTCCTTTTGCGAAAA | CGGCCAACGCCAAAACT | | |
| GAPDH promoter | TACTAGCGGTTTTACGGGCG | TCGAACAGGAGGAGCAGAGAGCGA | | EZ Magna Chip G kit |
| TNFSF15 | GCACCTCTTAGAGCAGACGG | TCGGCCTGCTTGTCTGATTT | | |
| SNCA | ACGACAGTGTGGTGTAAAGG | AACATCTGTCAGCAGATCTC | | |
| RSU1 | CATTGCAGACCAGTTCCAGC | GGGGTTTCCGGCTGATCTTT | | |
| MALAT1 | GTGATGCGAGTTGTTCTCCG | CTGGCTGCCTCAATGCCTAC | | |
| VEGFA | TTGCCTTGCTGCTCTACCTCCA | GATGGCAGTAGCTGCGCTGATA | | |

**Human probes for SCRINSHOT**

| | |
|---|---|
| MEG8_h_pr1_Cy3 | CCT GAG UTG GAA UCC UGG G[CY3] |
| MEG8_h_pr2_Cy3 | CTC CAT CUA TGC ATG UAT CTT GAT GUA GG[CY3] |
| MEG8_h_pr3_Cy3 | GAT CTU CAC CAG AAG ACT ATA AUT AAA TCA GGU A[CY3] |

| Antibodies for IP and ChIP | Species | Concentration | Manufacturer |
|---|---|---|---|
| H3K27me3 | mouse | 1 µg | Abcam ab6002 |
| EZH2 | rabbit | 5 µl | Active Motif 39901 |
| RNA polymerase II | mouse | 1 µg | EZ Magna Chip G kit |
| normal mouse IgG | mouse | 1 µg | EZ Magna Chip G kit |
| normal rabbit IgG | rabbit | 1 µg ChIP/5 µl IP | CST 2729 |

**Sequences for knockdown**

| Compound | Sequence or catalogue number | Manufacturer | |
|---|---|---|---|
| Negative control A | 339515 LG00000002-DDA | Exiqon | |
| MEG8 Gapmer | CAGACCTGACATCCAT | Exiqon | |
| MEG8 siRNA | GGAAUAGACGAGAUUGGAU | Sigma Aldrich | PMID:31711641 |
| MEG8 siRNA-as | AUCCAAUCUCGUCUAUUCC | Sigma Aldrich | |
| Mission siRNA universal negative control | SIC001-10NMOL | Sigma Aldrich | |
| TFPI2 siRNA | SASI_Hs01_00169244 | Sigma Aldrich | |

| Antibodies for Western blot | Species | Dilution | Manufacturer |
|---|---|---|---|
| TFPI2 | rabbit | 1000 | Abcam ab186747 |
| VEGF | rabbit | 500 | Proteintech 19003-1-AP |
| β-tubulin | rabbit | 1000 | CST 2128 |
| GAPDH | rabbit | 1000 | CST 2118 |
| HRP rabbit secondary | rabbit | 5000 | Dako P044801-2 |

**Supplementary table s2**
**Donor information (adapted from Pham et al. Frontiers in Cell and developmental biology 2020)**

| Patient | Group | Age | Gender | Patient information |
|---|---|---|---|---|
| 4062 | D | 55 | M | Grade IV subarachnoid haemorrhage. Xs inotropes; bad coron aa; CVD? Hi WBC drugs coron aa data |
| 3141 | D | 52 | M | Intercerebral haemorrhage (ICH) |
| 3145 | D | 39 | M | Motor vehicle accident (MVA) |
| 4095 | D | 48 | F | Grade V subarachnoid haemorrhage (SAH) |
| 4104 | D | 59 | F | LA/RA/RV; Cor aa |
| 4091 | ISHD | 53 | M | Heart failure 5 years; Renal failure,Dx 5years, 3x CABG, psorisis; AF; elevated cholesterol; plmonary hyperplaysia,LAD 100%;Graft 70%;LCX 50%;RCA graft 100% |
| 4093 | ISHD | 55 | M | Coronary artery bypass graft (CABG) 3x; LV 6y; hypothyroid;diabetes; AF;family history. MYHA Class III,AF,LV systolic impairment. FHx |
| 5068 | ISHD | 49 | M | Global ischemia, multiple stents |
| 4074 | ISHD | 61 | M | CABG 3x; Circumfl 70%, smoked 12 years,social drinker,angioplasty, multiple adhesions. Drugs: warfrin, amiodarome,digoxin,fusemide,temazapan, puride |
| 4108 | ISHD | 62 | M | CABG; ICD implant; IHD,CCF |

**Supplementary figure 4**

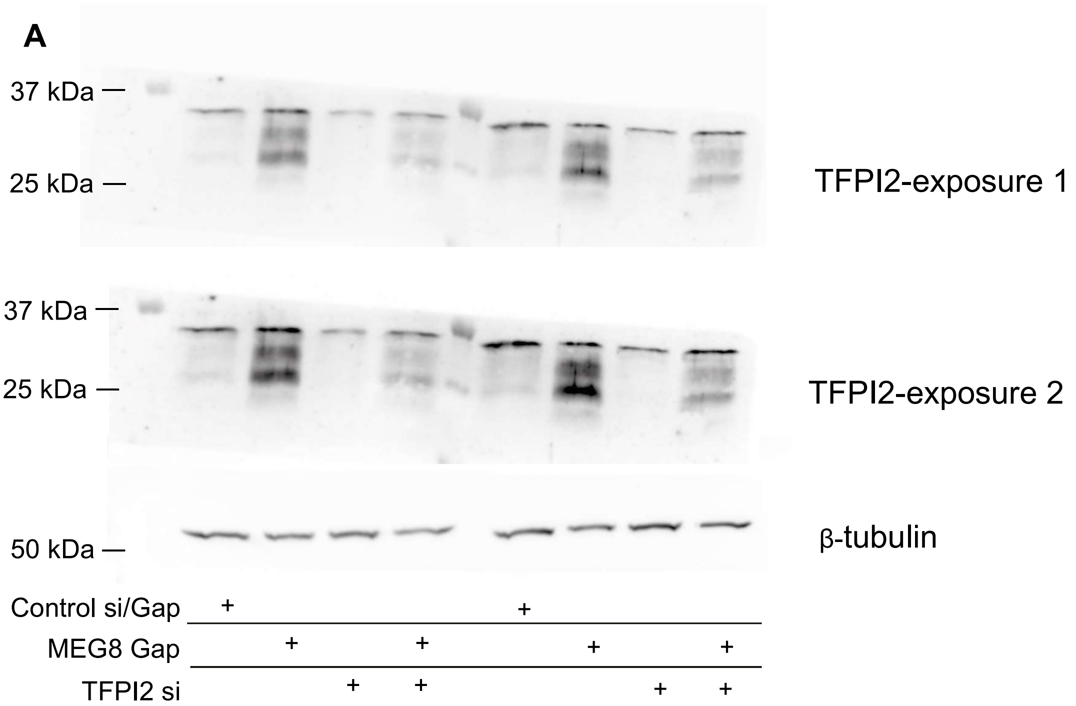


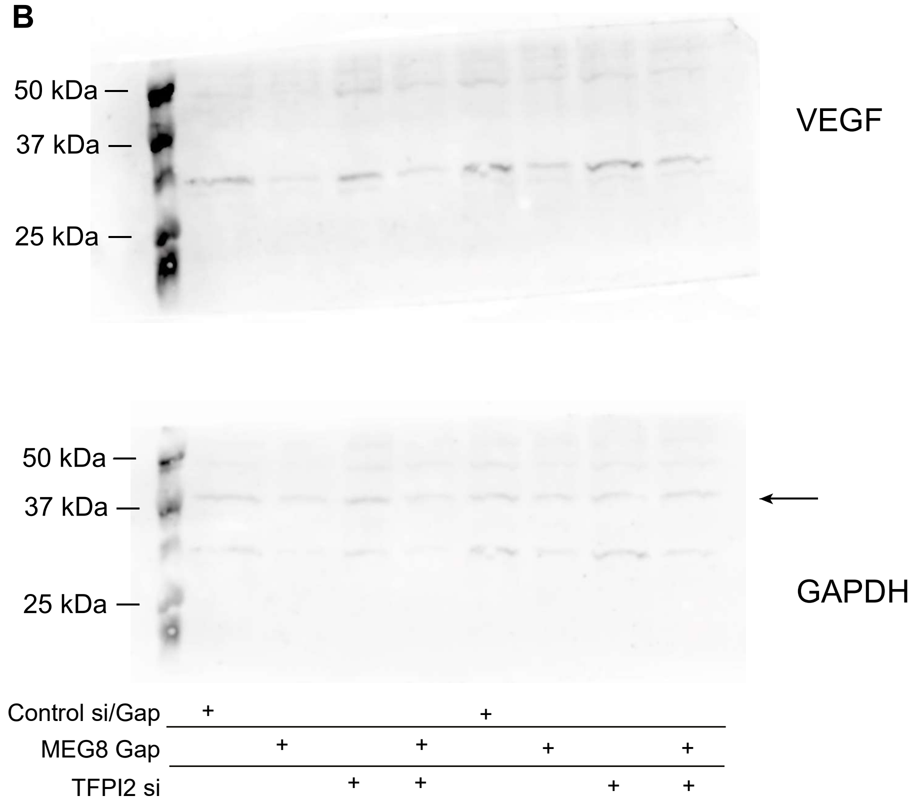

## Figure legends

**Supplementary figure 1**: **A**: Coding Potential Assessing Tool (CPAT) is used to evaluate coding potential of MEG8. MALAT1 and XIST are included as controls of non-coding transcripts. DLK1, DIO3 and RTL1 are coding transcripts found on chromosome in close proximity to the 14q32 non-coding cluster. **B**: The effect of MEG8 knockdown on migration was assessed by ECIS. After a monolayer had been established, an electrical pulse was applied to create a cell free area. Coefficient of the slope was calculated in the linear section. There is no significant difference between the slopes. **C**: Migration was also assessed by a mechanical scratch. The distance covered is presented as length in µm.

**Supplementary figure 2**: **A**: EC spheroids were embedded in collagen gels 24h after transfection with siRNA and stimulated with VEGF (10 ng/mL). Cumulative sprout length was determined by measuring the distance from the base of the spheroid to the tip cell. Groups were analysed using one way ANOVA. **B**: Proliferation was measured by EdU incorporation over 24 hours. The percentage of proliferating cells is shown. Groups were anaysed using unpaired t-test. **C**: VEGF expression after GapmeR-mediated silencing of MEG8 were analysed by RT-qPCR. RNA was collected 48 hours after transfection. Expression is relative to RPLP0. Groups were analysed using unpaired t-test. **D**: VEGF protein levels were determined using Western blot. Cell lysates were collected 48 hours after transfection. GAPDH was used as a loading control. Contrast was enhanced equally across the image for clarity. **E**: Changes in gene expression after silencing of MEG8 were analyzed by RNA sequencing. The z-score for the top 50 differentially expressed genes is shown. **F**: TFPI2 expression was measured by RT-qPCR in the left ventricle of ischemic heart disease patients and controls. Expression is relative to RPLP0. Groups were analysed using unpaired t-test. **G**: HUVECs were transfected with Control or MEG8 GapmeRs for 24 hours and exposed to 1 % hypoxia or normoxia for 24 hours. RNA was collected and gene expression was measured by RT-qPCR. Expression is relative to RPLP0. Data is presented as mean ± SEM. Groups were analysed using one-way ANOVA. Significance was indicated as: * $p < 0.05$, **$p < 0.01$, ***$p < 0.001$.

**Supplementary figure 3**: **A**: Subcellular localization of MEG8 was analyzed by SCRINSHOT RNA FISH. Nuclei and membrane were immunostained with DAPI. Separate channels are shown. Scale bar indicates 10 µm. **B**: MEG8 expression after GapmeR/siRNA-mediated silencing of MEG8 and TFPI2 were analysed by RT-qPCR. RNA was collected 48 hours after transfection. Expression is relative to RPLP0. Groups were analysed using unpaired ANOVA. Data are presented as mean ± SEM. Unpaired t-test was performed to compare the two groups. Significance was indicated as: * $p <0.05$, **$p < 0.01$, ***$p < 0.001$.

**Supplementary table s1**: List of reagents. RT-qPCR primers, Gene silencing and RNA SCRINSHOT oligo sequences. Antibody list used for Western blots and RNA IPs and ChIP.

**Supplementary table s2**: Patient information regarding ischemic heart disease patients (ISHD) and controls (D).

**Supplementary figure 4**: Original full size Western blots. **A**: TFPI2 protein levels were determined using Western blot. Cell lysates were collected 48 hours after transfection. β-tubulin was used as a loading control (Figure 2C in manuscript). **B**: VEGF protein levels were determined using Western blot. Cell lysates were collected 48 hours after transfection. GAPDH was used as a loading control. VEGF signal is also visible on GAPDH image (Supplementary figure 2D in manuscript). Secondary antibodies (Dako) were incubated for 2 hours at room temperature. Bands were visualized using enhanced chemiluminescence (ECL, Amersham/GE-healthcare) on the AI600 (Amersham/GE-healthcare).
